# Pityriasis rubra pilaris presenting with an abnormal autoimmune profile: two case reports

**DOI:** 10.1186/1752-1947-3-123

**Published:** 2009-11-13

**Authors:** Stamatis Gregoriou, Zoe Chiolou, Christina Stefanaki, Niki Zakopoulou, Dimitrios Rigopoulos, George Kontochristopoulos

**Affiliations:** 1 University of Athens Medical School, Department of Dermatology, Athens, Greece; 2 2nd Department of Dermatology, Andreas Sygros Hospital, Athens, Greece

## Abstract

**Introduction:**

Pityriasis rubra pilaris is an uncommon inflammatory and hyperproliferative dermatosis of juvenile or adult onset. The etiology of the disease is still unknown.

**Case presentation:**

We present the cases of two Caucasian men aged 53 and 48 who presented with pityriasis rubra pillaris type 1; both patients also exhibited an abnormal immunological profile.

**Conclusion:**

Pityriasis rubra pillaris is currently classified as a keratinization disorder. The abnormal immunological profile reported in our patients along with the comorbidity of pityriasis rubra pilaris with autoimmune disorders reported in the literature poses the question of a possible pathogenetic role for the immune response in this disorder.

## Introduction

Pityriasis rubra pilaris (PRP) is an uncommon hyperkeratotic, papulosquamous disease, classified into five groups subject to clinical appearance, age of onset and prognosis [1]. Recently, a sixth group has been proposed in acknowledgment of the HIV-associated type of PRP. The etiology of the disease remains unknown but several studies have reported an association of PRP with other autoimmune disorders [2-4]. We present the cases of two patients with type 1 PRP who presented with abnormal autoimmune profiles.

## Case presentation

### Case report 1

**Figure 1:**
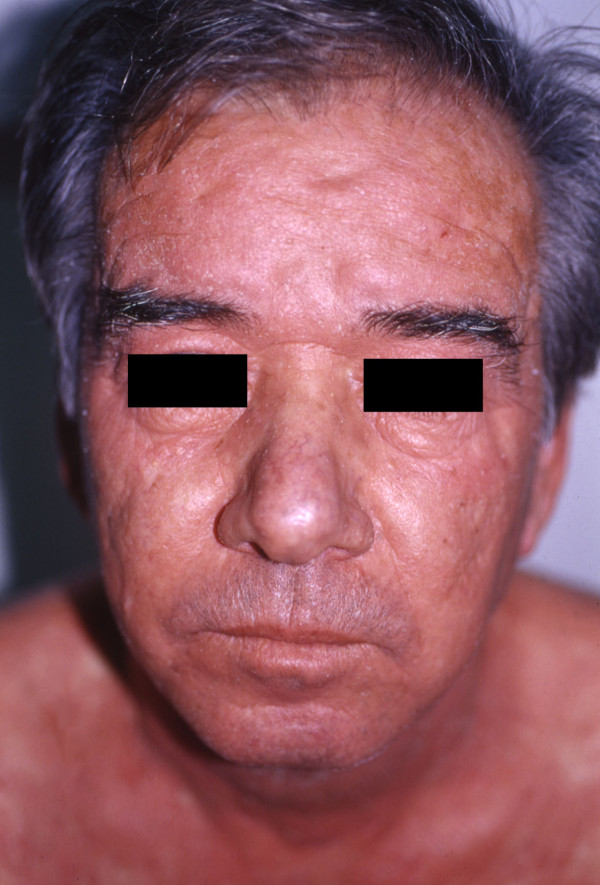
**Pityriasis rubra pilaris on the face of the first patient**.

**Figure 2:**
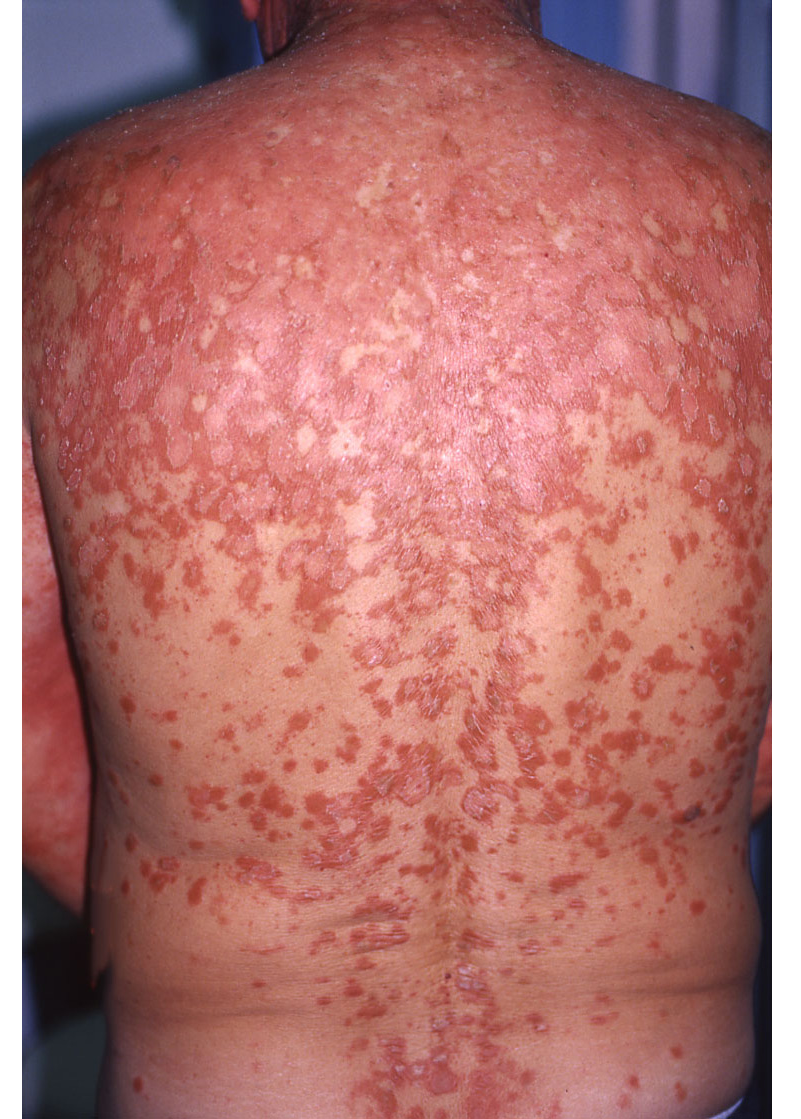
**Hyperkeratosis, parakeratosis and acanthosis in the epidermis of the first patient**.

**Figure 3:**
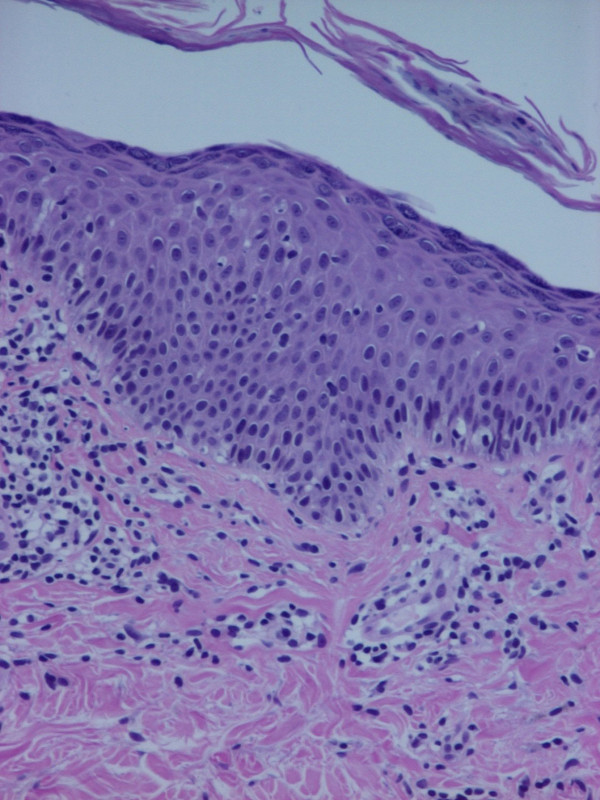
**Lymphocytic infiltrate in the dermis (hematoxylin and eosin, ×250)**.

A 53-year-old Caucasian man presented with a two-week history of slightly scaly pruritic erythematous plaques with an orange hue that covered his face (Figure 1), the extensor aspects of his arms, forearms and legs, upper trunk, buttocks and flexures. Patches of normal skin were evident within those sheets of erythema, together with prominent erythematous follicular papules at the margins of the plaques. His palms and soles were slightly hyperkeratotic with a yellowish hue. His past medical history was unremarkable. He had no arthritis, did not report symptoms or present with clinical signs that could be attributed to any autoimmune disorder. The results of his complete blood count, urine analysis and blood chemistry profile were unremarkable. Initially, antinuclear antigens (ANA) were weakly positive (1:80), later rising to high titers (1:1280) and showing a speckled pattern, whereas anti-DNA, extractable nuclear antigen (ENA), anticardiolipin antibodies and cryoglobulins were negative. C3 and C4 were mildly elevated, but CH50 was normal. The patient did not report any recent infection. Histopathology showed orthokeratosis alternating with parakeratosis, a normal granular layer, an absence of Munro microabscesses and dilatation of the dermal blood vessels with a low-grade perivascular inflammatory infiltrate (Figure 2, Figure 3). Both the clinical and histological pictures were compatible with PRP and the patient was commenced on acitretin 50 mg/day. Within 1 month, he had improved remarkably and his skin had become almost clear. His ANA titer had decreased to 1:640 after treatment.

### Case report 2

**Figure 4:**
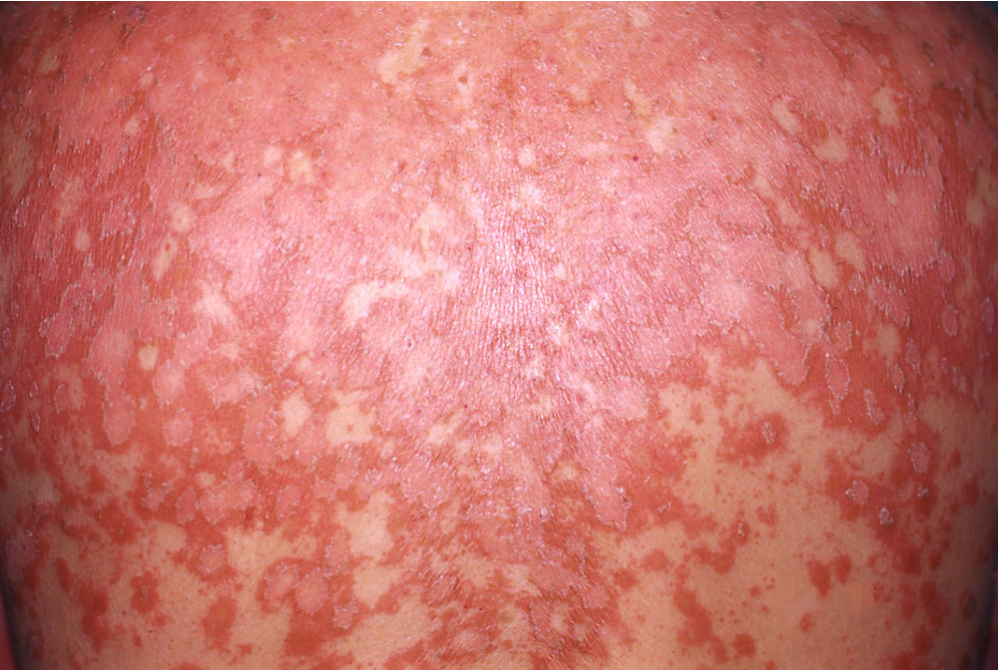
**Pityriasis rubra pilaris covering the back of the second patient**.

A 48-year-old Caucasian man presented to our clinic with a one-month history of pruritic, slowly expanding scaling lesions over his face, scalp, upper trunk and the outer aspects of his arms (Figure 4). His medical history was significant for coronary disease and diabetes mellitus II. He had no arthritis, did not report any symptoms and clinical examination did not reveal signs that could be attributed to any autoimmune disorder. Physical examination revealed slightly scaling erythematous lesions over his forehead, proximal anterior scalp, the nape of his neck, face, forearms and upper trunk. The results of his complete blood count, urine analysis and blood chemistry profile were unremarkable. His ANA displayed a speckled pattern and had an initial titer of 1:640 (negative >1:80) which decreased to 1:80 positive during therapy. Ro (Sicca syndrome A; SSA) antibodies were intensively positive (145, 1 U, negative <20) and La (Sicca syndrome B; SSB) antibodies were slightly positive (33 U, negative <20). C3 was mildly increased (223 mg/dL, normal: 84, 1-167 mg/dL), whereas C4 and CH50 were normal. Anti-dsDNA, anti-RNP, pANCA, cANCA anti-Sm antibodies, as well as antibodies against histones and antibodies against cardiolipin were not identified. Direct immunofluorescence from a sun-exposed lesion did not show immunoglobulin or complement deposition. The patient did not report arthralgias, myalgias or symptoms of any other system. According to his immunological profile and his clinical presentation, the patient was initially diagnosed as suffering from subacute cutaneous lupus erythematosus (SCLE). He was started on hydroxychloroquine 200 mg per day, but the disease eruption extended to his trunk and lower extremities. Gradually, his soles and palms became intensively hyperkeratotic, salmon-colored and he developed ectropion in both eyes. The histological findings of two biopsy specimens as well as his clinical picture were compatible with PRP showing alternate areas of orthokeratosis and parakeratosis. There was no atrophy of the epidermis. His hydroxychloroquine treatment was discontinued and he was prescribed acitretin 35 mg/day and prednisolone 40 mg daily tapered progressively. The lesions improved only temporarily. Methotrexate 25 mg per week intramuscularly was added and the patient improved remarkably within 3 weeks. He is still on follow-up. His SSA antibodies remained positive (130, 2 U, negative <20) on a subsequent check while SSB antibodies were within normal limits. C3 was slightly increased after treatment (247 mg/dL, normal: 84, 1-167 mg/dL).

A diagnosis of PRP was made for both patients based on clinical and histology data.

## Discussion

Case studies of PRP associated with autoimmune disorders including arthritis [2,5] and dermatomyositis [3] have been reported. Circumscribed juvenile-onset PRP was recorded in a patient with Down's syndrome and vitiligo [6]. PRP has also appeared simultaneously with SCLE, isolated IgA deficiency, hypogammaglobulinemia, hypothyroidism, celiac sprue, atopy, diabetes mellitus, underlying malignancy myasthenia gravis and therapy with agents known to disturb lymphoid function [4,5,7,8].

The first patient we describe had high titers (1:1260) of ANA, while the second patient's ANA displayed a speckled pattern and had an initial titer of 1:640 (negative >1:80) which decreased to 1:80 positive during therapy. Ro (SSA) antibodies were intensively positive (145, 1 U, negative <20) and La (SSB) antibodies were slightly positive (33 U, negative <20). C3 was mildly increased (223 mg/dL, normal: 84, 1-167 mg/dL). Boyd *et al. *have described a patient who presented with simultaneous PRP and SCLE and an ANA titer of 1:640 in a homogeneous pattern. Antibodies to dsDNA, histones, Smith antigen, Ro (SSA), La (SSB) and ribonucleoprotein were not identified. Moreover, a skin biopsy specimen from a sun-exposed lesion did not show immunoglobulin or complement deposition [4]. Another patient described by Polat *et al*. as having dermatomyositis displayed elevated serum creatine kinase and aldolase and rheumatoid factor but was negative for ANA, anti-DNA and anticytoplasmic antibodies [3]. Patients with PRP with joint and muscle involvement described in the literature had an unremarkable immunological profile [2,5].

The widely reported association of PRP with autoimmune disorders may point to an underlying abnormal immune response to antigenic triggers or microbial pathogens [7]. Investigators have reported an enhanced spontaneous activity of T-suppressor cells with an impairment of T-helper cell functions in a 6-year-old child with PRP [5]. The therapeutic efficacy of azathioprine and calcipotriol in PRP [7], both inhibitors of T-cell activation, as well as the association of PRP with T-helper cell dysfunction [7] and infections such as HIV [9] and hepatitis C [7] may support the hypothesis of this immunologic abnormality. Furthermore, patients with PRP have recently been managed with biological treatments including anti-TNF antibodies [10]. Given the response to anti-human TNF monoclonal antibodies, TNF-α may play a pathophysiological role in PRP.

## Conclusion

PRP is currently classified among keratinization disorders. A large meta-analysis of 26 PRP cases by Magro and Crowson that processed data from 250,000 dermapathology cases, revealed six cases with autoimmune coexistent disorders [7]. The authors conclude that a possible pathogenetic role for the immune response and its effect on epidermal retinoid signaling pathways warrants further investigation. Given the rarity of the disease, it is difficult to establish the precise association of PRP with autoimmune disorders. Nonetheless, we suggest that clinicians should look out for a coexisting autoimmune disorder or abnormal immunological markers in PRP patients.

## Patients' perspective

### Patient 1

I never had any problems with my skin. I suddenly developed a rash that kept spreading from my face downwards until it finally covered a significant part of my body. It was itchy at times. My hands were hard (hyperkeratotic) impeding the use of my fingers. I thought I had an allergy from food or garden work. I initially thought it would improve by itself using some over-the-counter products from my pharmacist but when it persisted I was really worried and went to the hospital. I was hospitalized in the dermatology clinic for 20 days and received a drug called Neotigason that greatly improved the problem and continued it at home. I'm fine now and still consult with my doctors at A Sygros hospital.

### Patient 2

This patient did not wish to contribute any comments.

## Abbreviations

ANA: antinuclear antigen; ANCA: antineutrophil cytoplasmic antibody; ENA: extractable nuclear antigen; PRP: pityriasis rubra pilaris; RNP: ribonucleoprotein; SCLE: subacute cutaneous lupus erythematosus; SSA: Sicca syndrome A; SSB: Sicca syndrome B; TNF-α: tumor necrosis factor alpha.

## Consent

Written informed consent was obtained from the patients for publication of this case report and any accompanying images. A copy is available for review by the Editor-in-Chief of this journal.

## Competing interests

The authors declare that they have no competing interests.

## Authors' contributions

GS, SC, and RD were the doctors who hospitalized the first patient and were responsible for diagnosis, laboratory results and therapy while CZ, ZN, and KG were the doctors responsible for the second patient. GS and KG were responsible for the design of the article proposing the possible association between PRP and immunological abnormalities. All authors have contributed in writing portions of the paper. All authors have read and approved the final manuscript.
